# Overview of the Current State of Gallium Arsenide-Based Solar Cells

**DOI:** 10.3390/ma14113075

**Published:** 2021-06-04

**Authors:** Nikola Papež, Rashid Dallaev, Ştefan Ţălu, Jaroslav Kaštyl

**Affiliations:** 1 Department of Physics, Faculty of Electrical Engineering and Communication, Brno University of Technology, Technická 2848/8, 61600 Brno, Czech Republic; nikola.papez@vutbr.cz (N.P.); xdalla03@stud.feec.vutbr.cz (R.D.); 2 Directorate of Research, Development and Innovation Management (DMCDI), Technical University of Cluj-Napoca, Constantin Daicoviciu Street, No. 15, 400020 Cluj-Napoca, Romania; 3 Central European Institute of Technology, Purkyňova 656/123, 61200 Brno, Czech Republic; jaroslav.kastyl@ceitec.vutbr.cz

**Keywords:** gallium arsenide, solar cells, structure, application, degradation, space, concentrators, uav

## Abstract

As widely-available silicon solar cells, the development of GaAs-based solar cells has been ongoing for many years. Although cells on the gallium arsenide basis today achieve the highest efficiency of all, they are not very widespread. They have particular specifications that make them attractive, especially for certain areas. Thanks to their durability under challenging conditions, it is possible to operate them in places where other solar cells have already undergone significant degradation. This review summarizes past, present, and future uses of GaAs photovoltaic cells. It examines advances in their development, performance, and various current implementations and modifications.

## 1. Introduction

Gallium arsenide is a material widely used mainly in semiconductor technologies due to its attractive properties, where it has found many uses. In contrast to silicon, it has become very popular in high electron mobility transistor (HEMT) structures since it does not require any momentum change in the transition between the maximum of the valence band and the minimum of the conductivity band, and does not require a collaborative particle interaction. However, hole mobility, in contrast to much higher electron mobility, is similar to silicon—the response times are the same for devices that require cooperation between the motion of holes and electrons. The direct bandgap of GaAs of 1.42 eV is also suitable for diode and photovoltaic (PV) cell applications. It is often extended by so-called alloying, i.e., precise melting of two elements together, in this case, with aluminum, to give Al${}_{x}$Ga${}_{1-x}$As. The advantage of a wide bandgap is also the fact that the material remains more semiconductive at higher temperatures, such as in silicon, which has a bandgap of 1.12 eV. With higher temperatures, the thermal generation of carriers becomes more dominant over the intentionally doped level of carriers [1,2]. Therefore, GaAs solar cells have also become the standard for use in demanding temperature conditions. The production of wafers is generally more difficult and expensive. Due to the temperature gradient acting as mechanical stress, more crystalline defects are created: a standard diameter of 6″ wafers is used compared to 12″ for silicon [3]. Single crystals of GaAs are very brittle. Germanium is often used as a substrate, which is suitable for its high mechanical strength and atomic lattice spacing very similar to GaAs [4].

GaAs PV cells belong to III–V group compounds, according to the newer IUPAC notation, already referred to as groups 13–15. Nonetheless, Roman numerals are still familiar, which means this is a semiconductor compound of at least two chemical elements. In 2000, a significant contribution to GaAs was credited to the Nobel prize-winning Russian physicist Zhores Alferov in the field of heterostructures [5].

For GaAs-based solar cells, performance can also be tuned by layering, where one solar cell can contain up to eight thin layers, each absorbing light at a specific wavelength. Such photovoltaic cells are called multi-junction or cascade solar cells. They use tandem fabrication, so they can also be found under the name tandem cells. Each layer contains a different composition and material with a specific bandgap that absorbs light in a particular spectral region. Usually, the top layer has a large bandgap and absorbs most of the visible spectrum up to the bottom layer with a low bandgap, which absorbs light in the infrared region [6]. By covering a wide spectral electromagnetic range, maximum efficiency can be achieved. Other layers are commonly used, such as GaAs, AlGaAs, InP, InGaP, and GaInAs. Due to the mentioned mechanical strength and oriented growth of the Ge crystal lattice, it is possible to make very thin layers, reducing the overall weight of the PV cell.

Multi-junction solar cells, or thin-layer solar cells are referred to as the second generation of solar cells, which has also already been successfully commercialized. It is, therefore, not an experimental technology but a very mature and mastered technology that is already used in many areas. Thanks to such a multi-layered construction, they achieve higher efficiency than conventional single-layer solar cells. In March 2016, Yamaguchi et al. developed the triple-junction PV cell with 37.9% efficiency under 1 Sun, and 44.4% efficiency together with concentrator under 246–302 Suns [7]. In April 2020, a study was published in Nature Energy [8], where the authors of the six-junction PV cell achieved an efficiency of 39.2% and a value of 47.1% at 143 Suns, using the concentrator which was also certified by NREL. They also claimed that further reduction in the limiting series resistance should result in efficiencies over 50%.

**Figure 1:**
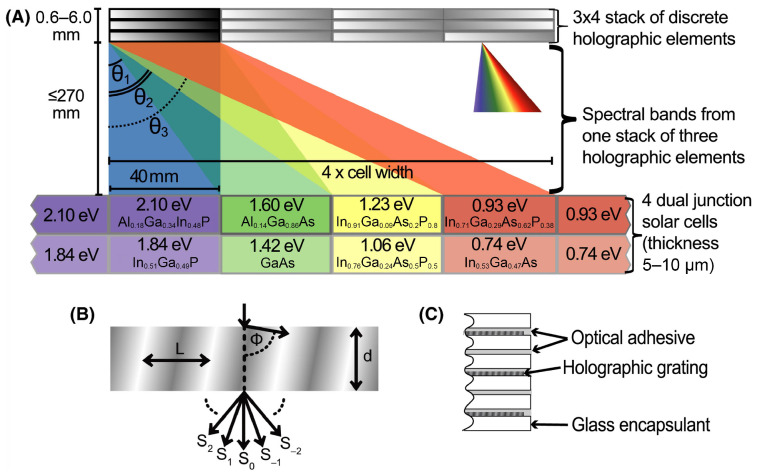
(**A**) Stacks of discrete holographic elements (a single stack is described in part (**C**)) generate four spectral bands coupled into one of four dual-junction solar cells, including GaAs. Part (**B**) shows the volume phase hologram of thickness *d* with fringes representing the refractive index with periodicity *L*, tilted to the grating normal by angle ϕ, where incident light is split into diffracted orders $S_{i}$ [9].

Another interesting use of cells was the design of the first holographic diffraction system to incorporate eight solar subcells, more precisely, four different dual-junction PV cells, as can be seen in Figure 1. Darbe et al. declared by simulations 33.2% module conversion efficiency, including external losses, and 63.0% with ideal cells and optics [9].

The most common field using GaAs-based solar cells is the aerospace industry [10,11]. The main reason is their wide spectral coverage, which is much larger in space than on Earth. They are also used in the aviation and military due to their flexibility and weight, which can be used especially for unmanned aerial vehicles (UAVs); and last but not least for concentrators, thanks to which solar cells can operate at very high temperatures. However, from a practical point of view, this type of solar cell is expensive for common use. Prices may vary depending on the complexity of the technology—the number of junctions. The high price is influenced not only by the cost of the wafer but also by subsequent production—expensive equipment. Li et al. state that compared to silicon, the prices of GaAs cells are up to ten times higher [12]. In contrast, the prices of silicon cells are very affordable today. Since 1977, when the cost per watt was around 76 dollars, it is now approximately 36 cents [13].

## 2. Structure and Composition of GaAs Solar Cells

As mentioned in the introduction, not only have single-junction solar cells been developed for a long time, but multi-junction structures are being created to achieve the highest possible performance. The composition of these structures depends on the specific use. Thus, it is clear that, for example, the light of a different spectral range than on Earth will fall on the surface of Mars due to its atmosphere. Therefore, the Earth’s atmosphere filters not only harmful radiation for humans but also radiation that the solar cell can use. For multilayer structures, emphasis is placed on high crystal perfection in order to avoid recombination of generated minority carriers at cracks and other defects [14,15]. By default, production takes place by growing on a doped substrate. The specific substrate is chosen depending on the next layer that will grow on it to induce an ideal lattice within the epitaxy. The most typical materials are described in Table 1.

As shown in Table 1, temperatures at 300 K or even at 0 K are standardly presented. If necessary, the bandgap at any temperature can be determined empirically by fitting according to the Equation (1) by Varshni [18,19]:

(1) $$E_{g}\left(T\right)=E_{g}\left(0\right)-\frac{\alpha T^{2}}{T+\beta },$$

where *T* is the desired temperature in Kelvin, $E_{g}\left(0\right)$ is the energy gap at 0 K of a given semiconductor, and α and β are specific material constants. So, if we want to calculate the GaAs energy bandgap, for example, at 273.15 K, where $E_{g}\left(0\right)=1.52$ eV, α=0.5404meV/K and β=203K, the result would be:

(2) $$E_{g}\left(273.15\right)=1.52-\frac{0.5404\cdot 10^{-3}\cdot 273.15^{2}}{273.15+203}=1.435\mathrm{eV}.$$

Well-established epitaxial crystal growth techniques include metal–organic vapor phase epitaxy (MOVPE) and molecular beam epitaxy (MBE). Both methods originated in 1960 and have some differences [20].

- MOVPE is used to deliver faster growth rates for bulk layers and low breakdown at high temperatures and low vacuum. MOVPE does not require significant bake times and can recover more quickly from equipment failures than MBE.
- MBE is, unlike MOVPE, considered a method for superior quality and pure materials in ultra-high vacuum (UHV). It is easier to maintain and is able to grow thermodynamically forbidden materials [21].

There are also several grown concepts that can even be combined, as mentioned, for example, in the inverted metamorphic (IMM) solar cell in Section 3.3. This structure is currently relatively frequently used.

- Lattice matched [22]
- Upright [23]
- Metamorphic [24]—use the localization of defects in a buffer layer located between layers with different lattice constants.
- Inverted [25]—this is an inverted growth of the structure, so materials with a higher bandgap grow here first. The structure is then rotated, and the substrate is removed. This leads to a better performance of the solar cell.

After the growing process, the solar cell is finished by layer bonding, an anti-reflection coating (ARC), and contact metallization [26]. Very thin contacts in the range of micrometer units are often used.

## 3. Applications of Solar Cells

As mentioned in the introduction, GaAs and multi-junction PV cells are used mainly in particular industries, where they are required to be highly efficient, durable, or lightweight. These are cutting-edge technologies for special purposes.

### 3.1. Aerospace and Military

**Figure 2:**
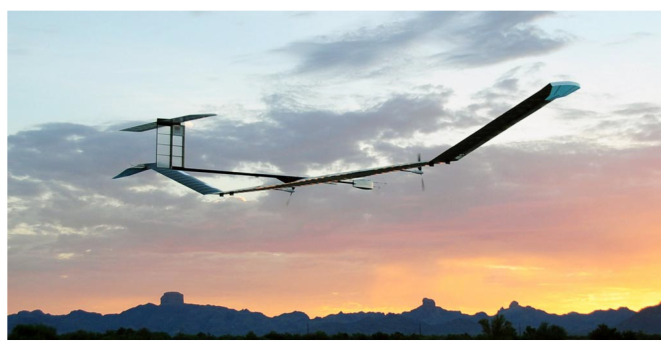
Airbus Zephyr during flight [34].

Experimental high-altitude long-endurance UAVs are aircraft that are covered mainly with flexible solar cells because of stay in the air for up to months. They thus replace launching satellites into orbits, which are usually covered by considerable expenses. UAVs can then serve for mapping, surveillance, border patrol, or search and rescue. For civilian use, they are used in flying cell phone towers and communications. Experiments with UAVs and solar cells have been around for over 20 years, and there is constant progress [27,28,29]. Recent advances have been made since 2017 by Alta Devices, where their flexible solar cells exceed efficiencies of 30%, aerial densities of 170 g/m, and are 30 μm thick. Their solar cells are widely used for aerospace purposes [30]. Microlink Devices Inc. also supplies solar cells to the UAV sector. For example, for Airbus Zephyr (Figure 2)—a solar high-altitude platform station operating in the stratosphere with >29% AM0 efficiency [31,32]. Last but not least is the Thales Stratobus airship capable of flying at an altitude of 20 km, which previously used a transparent envelope section that allows sunlight reflection in concentrator mirrors, which were directed to solar arrays inside the UAV. However, since 2018, this system has been abandoned and replaced by flexible multi-junction arrays installed on the top surface [33].

**Figure 3:**
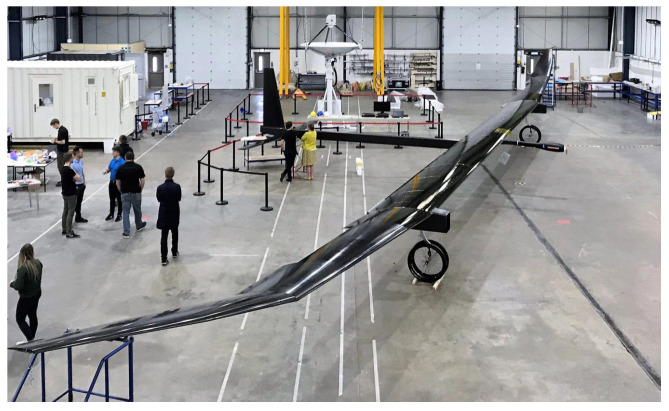
Prepared UAV PHASA-35 in hangar built by Prismatic for BAE Systems [43].

It is also worth mentioning other areas where flexible multilayer panels are, or have been, in use. These include Aquila by Facebook (discontinued) [35,36], Solara 50 by Google, formerly Titan Aerospace (discontinued) [37], HAWK30 by AeroVironment Inc. [38], Caihong (Rainbow) T-4 by the Chinese Academy of Aerospace Aerodynamics [39], PHASA-35 by BAE Systems (Figure 3) [40], Odysseus by Aurora Flight Sciences [41], etc. Even though GaAs flexible cells are constructed for most UAVs, these projects for the long-term sustainability of aircraft in the air are very demanding and have been evolving for a long time. Most of them are in experimental phases. In addition to Alta Devices, Sharp Corporation and SolAero Technologies Corp. are other significant manufacturers producing multilayer solar panels [42].

### 3.2. Solar Photovoltaic Concentrators

Together in the combination of GaAs PV cells, solar concentrators are widely used, i.e., devices consisting of various optical elements that concentrate light, most often sunlight, into one central point, which is a solar cell. Concentrator photovoltaics (CPV) are used to express the intensity of concentration in the number of Suns or ratios. By default, if the light intensity on the solar cell exceeds 10 Suns, it is already necessary to use passive cooling of the PV cell. This system is considered a low-concentration photovoltaic system (LCPV), and silicon solar cells can still be used here. If the light intensity exceeds 100 Suns, the solar cell must already be actively cooled by cooling fluid, and in that case, it can be considered high-concentration photovoltaics (HCPV). This is a nearly relative number and varies in the literature. GaAs and multilayer structures are already used exclusively for such performance concentrators.

Many concentrator designs follow the concept of Fresnel lens, reflectors, parabolic mirrors, or luminescent concentrators. Notwithstanding, it always depends on their use. Kasaeian et al. summarized the parabolic and Fresnel-based photovoltaic thermal systems over several years, where GaAs cells have always given excellent performance compared to other conventional cells [44].

Solar cells, such as InGaP/GaAs/InGaAs inverted triple-junction, manufactured for the concentrator application, are also specially made for CPV, where Sasaki et al. achieved an efficiency of 45% [45]. In a similar way, concentrators can be created for a particular type of cell and used, for example, in space [46,47]. One such prototype was made by Warmann et al., which also served as ultralight multilayer optical coatings to increase the thermal emissivity of the concentrator and enhance radiative transfer. This unique parabolic concentrator was able to achieve a concentration of 15 Suns for the 1 mm wide cell [48].

**Figure 4:**
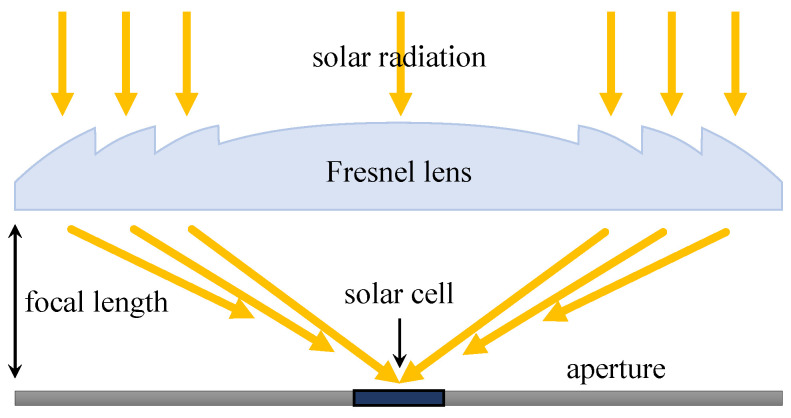
Fresnel lens concentrator focusing the light into one point without SOE [50].

One of the most applied and at the same time the oldest concentrators are Fresnel lenses, which are among the first concentrators to be used since 1979. Lenses are light and capable of achieving a short focal length and large aperture. They can be used in the construction in a shape of a circle focusing the light in a point like in Figure 4 (which is considered the most widespread) or in a cylindrical shape focusing the light in a line, resulting in a lower ratio concentration than in the previously mentioned construction. Their disadvantage is that the optical efficiency is limited by low or high temperatures and consequently by a change in the refractive index or deformation of the Fresnel structure by virtue of thermal expansion [49].

Application example of Fresnel lens optic made with Silicon-on-Glass (SoG) technology and designed by Fraunhofer ISE are FLATCON^®^ concentrator modules [51]. In 2003, the first module consisted of 16 cm${}^{2}$ lenses and GaAs single-junction solar cells in 2–4 mm diameter. Later, Wiesenfarth et al. performed ten years of outdoor measurements, where triple-junction solar cells were used. Long-term stability was observed when the efficiency per year decreased by (−0.25 ± 0.18)%_rel_ [52].

**Figure 5:**
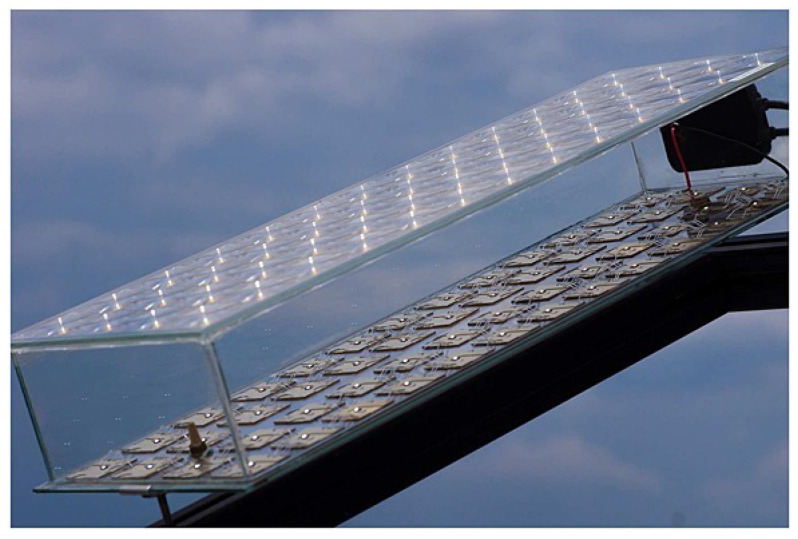
FLATCON^®^ CPV module with 52 four-junction solar cells [51].

Steiner et al. measured the performance of 52 four-junction solar cells using FLATCON^®^ modules (Figure 5) for one month under concentrator standard operating conditions (CSOC) and concentrator standard test conditions (CSTC). The rated efficiency was 35.0% at CSOC and 36.7% at CSTC, and were calculated as mean values [51].

**Figure 6:**
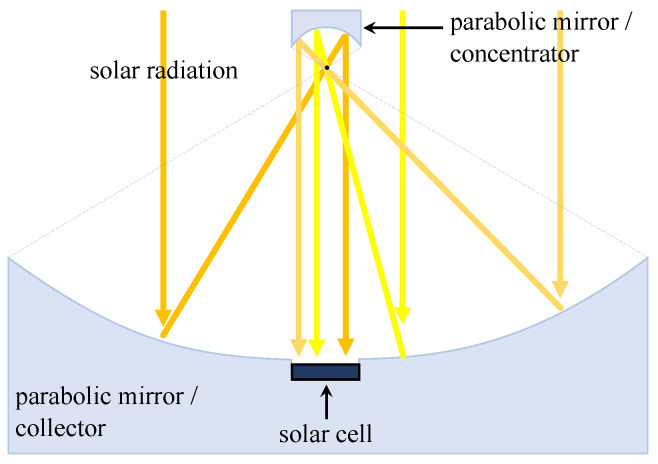
Parabolic mirror concentrator without optical lenses [50].

As another very popular concentrator type, and principally very powerful, where optical lenses are not used, is the parabolic concentrator [53]. It is usually utilized using two curved mirrors (Figure 6)—generally reminiscent of a parabolic antenna. The first larger mirror serves as a collector and the second as a focal point. However, various modifications exist where the focal point is already replaced by a solar cell. Like Fresnel lenses, they have a high ratio of around 500. These concentrators are often used in conjunction with thermal collectors (therefore, in the literature can be found for parabolic concentrators name collectors) and thus form a hybrid system. For example, in such a hybrid system, Widyolar et al. demonstrated the GaAs cell load of up to 365 ${}^{\circ }C$ with a thermal efficiency of around 37% [54]. More complex modern designs already count on a hybrid tubular thermoelectric generator, where the thermal model of the hybrid system with GaAs cells was studied [55].

**Figure 7:**
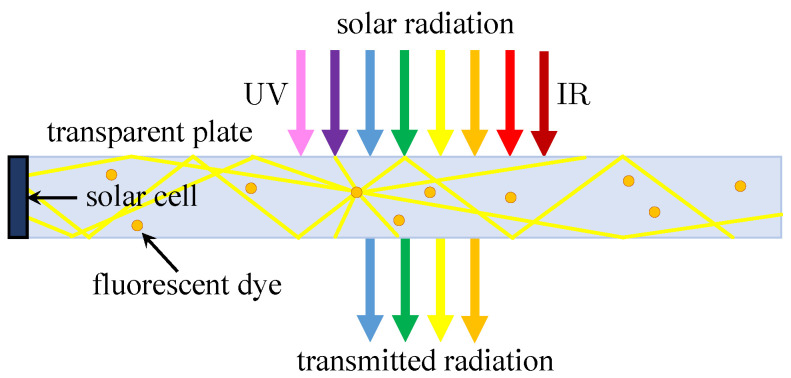
The basic construction of LSC with solar cell located on one side [50].

The opposite case of very powerful parabolic concentrators is luminescent solar concentrators (LSC), which are basically composed of one or more glass or plastic plates. The light captured in these plates, which serve as a waveguide, is guided to one or more edges by total internal reflection (light bounces around the material) where the solar cell is located (Figure 7). High performance is not expected here, but silicon solar cells, as a result of their small bandgap, are no longer adequate for these needs, and GaAs multilayer structures are used for acceptable performance. The plates contain fluorescent dye or quantum dots, so they emit absorbed light at longer wavelengths. Their ratio concentration factor can be up to 10 and they are used mainly as transparent and semi-transparent materials for covering buildings, or as solar windows. One such experiment was performed by Slooff et al., where multi-crystalline silicon (mc-Si), GaAs, and InGaP solar cells were investigated. The highest efficiency of 7.1% was achieved by GaAs solar cells when attached from four sides [56].

### 3.3. Probes, Satellites and Other Space Objects

Probably the most extensive use has been made of GaAs-based solar cells on space satellites, probes, and other objects, primarily because of the potential risk of gamma radiation, where GaAs also show higher resistance.

The first probes to carry GaAs-based solar cells were part of the Soviet Venera program used to explore the surface of Venus [57]. The probe Venera 2 was launched on 12 November 1965 and subsequently, after Venera 3 on 16 November 1965, from the Baikonur cosmodrome. Venus 3 is thought to have been the first human object to hit a foreign planet, but Leverington contradicts this claim due to a much earlier signal loss [58]. It is, therefore, uncertain whether the touchdown with the surface took place.

Another popular object using GaAs solar cells is the Hubble telescope, where the GaAs solar arrays with dimensions 7.1×2.6 m were installed in 2002 during Servicing Mission 3B. Solar panels replaced previous silicon ones [59].

Another exciting application is triple-junction solar cells by EMCORE Corporation for Orion Multipurpose Crew Vehicle (MPCV), which is a NASA spacecraft service module, and part of the Artemis 1 mission to travel around the Moon planned in November 2021 [60].

**Figure 8:**
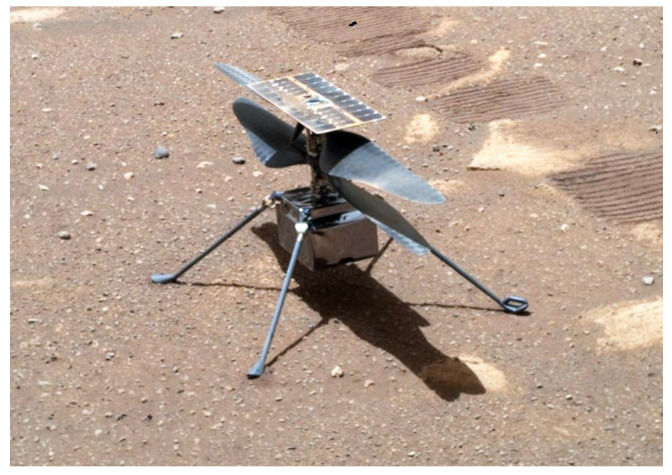
Image of the Ingenuity helicopter on Mars acquired on 7 April 2021 (Sol 46). IMM multi-junction solar cells are clearly visible from its top [64].

Many other solar system probes and other spacecraft utilize this type of solar cell and are active in space. Examples are the Venusian probe Akatsuki (InGaP/GaAs/Ge) [61], the robotic lander InSight (InGaP/InGaAs/Ge) to study the deep interior of Mars or the asteroid study probes Hayabusa2 and OSIRIS-REx [62]. Another current example is mission Mars 2020, which started at the end of July 2020. The Ingenuity helicopter (Figure 8) equipped with inverted metamorphic multi-junction solar cells specially tuned to Mars conditions by SolAero, which, together with the Perseverance rover, was part of the cruise stage. Its entire primary part, which was dropped just before the touchdown, was also covered by multi-junction GaAs solar cells. SolAero, which was mentioned in aeronautics applications, is a company that is also very involved in manufacturing and space applications [63].

Concentrators in space can also be used. However, there are some limitations. For example, near-Earth applications should use lower concentrations (5 Suns) in virtue of the more difficult heat dissipation [10]. However, concentrators in space have become very useful for far-Sun missions to increase low light intensities [65]. It is, hence, essential to know which light intensities can affect the cell.

#### Light Intensity Affecting Solar Cells in Space

In Earth’s orbit, the light intensity is $E_{s}=1367W/m^{2}$, which is equal to solar constant. The factor of decrease in flux is, therefore 4.62 × 10^4^ [66]. In the case of need to calculate the solar constant on Mars, the formula would be:

(3) $$S_{C}=\frac{L_{⊙}}{4\pi \;\cdot \;r^{2}},$$

where the constant $L_{⊙}$ is the solar luminosity of 3.828 × 10^26^ W and *r* is the distance of Mars from the Sun, which is 2.2794 × 10^11^ m. The solar constant on Mars would therefore be 586 W/m^2^ [67,68].

Because the Earth is in thermal equilibrium with this radiation equal to the solar constant, it must indeed emit the same amount. By adjusting this equality, we can approximate the effective temperature of the Earth as:

(4) $$\begin{array}{c} 4\pi R_{⊕}^{2}\sigma T_{⊕}^{4}=T_{⊙}^{4}\frac{R_{⊙}^{2}}{a_{0}^{2}}\pi R_{⊕}^{2}\\ \to T_{⊕}=T_{⊙}\sqrt{\frac{R_{⊙}}{2a_{0}}}\approx 279K, \end{array}$$

where $T_{⊕}$, $T_{⊙}$ and $R_{⊕}$, $R_{⊙}$ are the effective temperatures and radii of the Sun and the Earth, σ is the Stefan–Boltzmann constant, and $a_{0}$ is the distance of the Earth from the Sun [69].

Sunlight from the Earth is reflected or absorbed by the satellite and generates excess heat. The total irradiance $E_{\mathrm{ABS}}$ absorbed by the solar cell on the satellite can be calculated as follows:

(5) $$E_{\mathrm{ABS}}=T_{\mathrm{AR}}E_{S}\left(1-\eta \right)+A_{\mathrm{BULK}}\alpha \left(E_{S}+\sigma T_{⊕}^{4}\right),$$

where $T_{\mathrm{AR}}$ is the transmittance of the anti-reflective coating of the PV cell, η is the efficiency of the cell, $A_{\mathrm{BULK}}$ is the absorbance of the bulk cell, and α is the albedo of the Earth (a diffuse reflection of solar radiation from the Sun) [50].

## 4. Stability and Degradation of Structures

From the text above, it is clear that GaAs cells are used in devices where the emphasis is on considerable performance and stability. For probes, it is assumed that GaAs cells will no longer be serviced or changed. For HCPV systems, their operation is expected even under extreme conditions, as they are highly stressed by temperature. Among other things, these conditions occur in space, not only in high temperatures but also in low temperatures.

An extensive study using several methods on a single-junction GaAs cell was conducted by Papež et al., which dealt with the degradation of GaAs cells over the past few years. Degradation after thermal processing [70,71], after cooling [6], after exposure to gamma radiation [50,72], and after exposure to broadband radiation was studied [73]. An unstressed sample was also observed, and defects and contamination after fabrication were examined [74].

**Figure 9:**
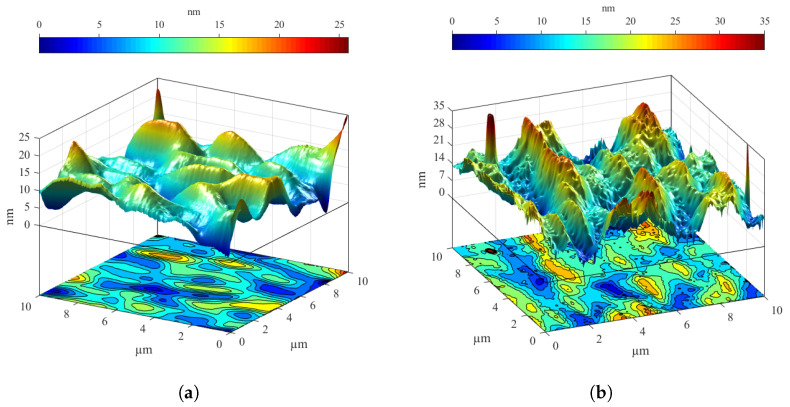
The differences (**a**) before and (**b**) after thermal processing of the cell scanned by an atomic force microscope (AFM) are considerable. The surface structure is entirely different. The average height of the feature on the surface changed from 7.16 nm to 15.73 nm after thermal heating [70,76].

During thermal heating, the samples were kept at 350 ${}^{\circ }C$ for 240 min. The measurement was performed even with a short-term 30 min stress, when a temperature of up to 420 ${}^{\circ }C$ was chosen. In both cases, the samples were shown to be functional, but the decrease in performance was noticeable, which can be seen from several parameters in Table 2. At a temperature of 420 ${}^{\circ }C$, there was already a considerable failure rate, and this could be considered a short-term limit value. The occurrence of surface defects and an evident change in morphology were obvious in Figure 9. However, it can be expected that the loss of solar cell performance is not only caused by a different surface structure but also by internal degradation processes [70,71,75].

On the contrary, after cooling in vacuum up to −120 ${}^{\circ }C$, the changes on the GaAs-based PV cell surface were also measured in the form of reflectance. Reflectance was measured outside the vacuum chamber, where minimal differences were observed. It was mentioned that a significant decrease in the power of the solar cell could be affected by a negative thermal coefficient [6].

**Figure 10:**
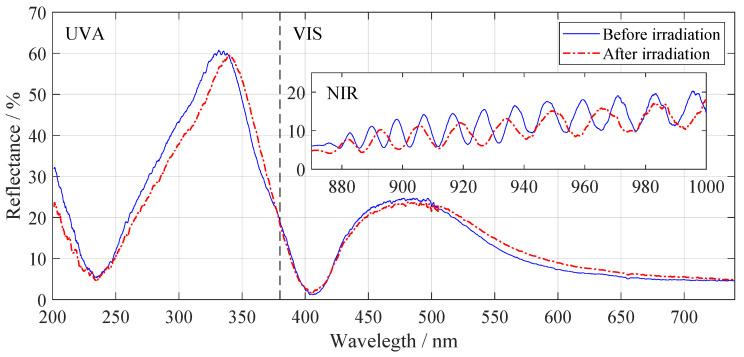
The figure shows the reflectance before and after irradiation, divided into three groups—the ultraviolet spectrum, the visible region, and the near-infrared region. No significant changes are observed in the first two sections mentioned. Noteworthy is the last near-infrared region, where interference fringers give us information about changes in the thickness of the top layers [72].

Papež et al., also in 2020, extensively studied the degradation of cells depending on gamma rays irradiation using a Cobalt-60 emitter when a dose of 500 kGy was applied. The measurements took place within the electrical, optical, chemical, and structural characterization framework, which complemented each other. After a high irradiation dose, the solar cell worked without problems, but the efficiency decreased (fill factor decreased from 0.72 to 0.48). In addition to changes in morphology, it was discovered that after irradiation, elements that are part of the ARC diffused deeply into the material. The difference in the top thin layers is indicated in Figure 10. This phenomenon could cause a loss of cell performance [50,72]. Other extensive studies are underway by many authors on the radiation of either electrons [12,77] or protons [78,79].

**Figure 11:**
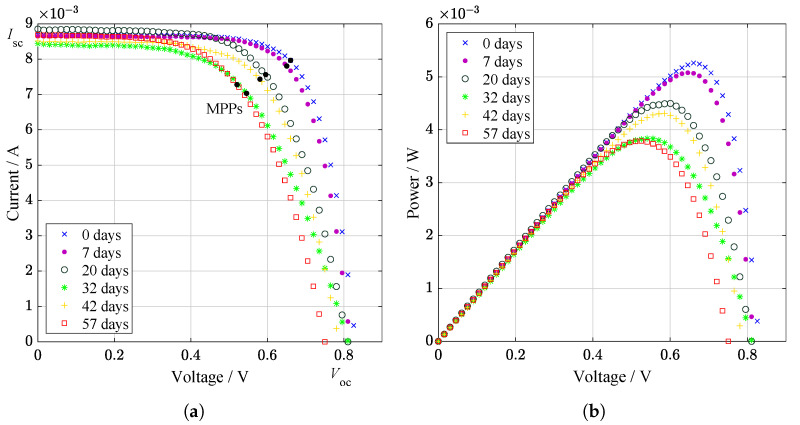
(**a**) Light I–V curves and recalculated (**b**) power characteristics of GaAs specimen under supercontinuum laser processing. Maximum power points (MPPs) are marked. On day 42, efficiency improvements can be seen [73].

Similarly, but on a smaller scale, Ti and Al atoms originating from anti-reflective layers migrated when the solar cell was spot irradiated with a supercontinuous laser with a power of 188 mW and a spectral range of 450 to 2400 nm. Here, the measurement was performed over a period of 67 days. The performance of the PV cell was also examined in real-time during the measurement of the sample. Interestingly, the degradation was not linear—there was a slight increase in efficiency at 42 days of irradiation in Figure 11, which could be due to the appearance of deep donor level centers (DX centers) [73]. The exact values from the measurement corresponding to Figure 11 are also added in Table 3.

**Figure 12:**
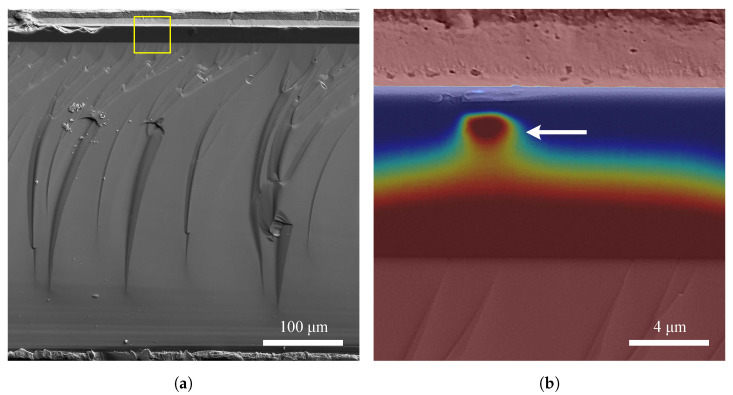
The images show cross-sectional view of the GaAs PV cell on a SEM microscope. The image (**a**) shows the complete structure of the PV cell. Contacts are visible from below and from top (contact is longitudinal along the edge). The largest part of the picture is occupied by germanium. However, the most important are the thin layers (the darkest part). The image (**b**) on the right represents the part marked with a yellow rectangle in image (**a**). The colored EBIC method (**b**) is used to visualize the distribution of carriers in the pn junction area. There is also applied bias voltage of −1 mV. Impurity (pointed by arrow), which was probably contaminated during the fabrication, is electrically active and allows easier tunneling of electrons through the junction [74].

Using the electron beam-induced current method (EBIC) Papež et al. also examined subsurface defects in the GaAs cell, where they found electrically active impurities affecting the pn junction during a cross-sectional view as illustrated in Figure 12. As the bias increased, there was gradual tunneling of electrons. However, this phenomenon did not have a permanent effect [74].

Many defects and impurities during imperfect fabrication can occur, and it is not always easy to eliminate them. It can be, for example, the fill factor and voltage loss caused by shunt or series resistance; interface recombination loss caused by lattice mismatching defects; bulk recombination loss caused by various defects, dislocations, and impurities; optical loss caused by poor ARC texture; or surface recombination loss caused by surface states. Thus, it is necessary to produce the best possible high-quality epitaxial growth, perfect lattice-matching layers, and ARC [80].

## 5. Conclusions

In this review, GaAs solar cells were discussed in many ways. In terms of use, their construction but also degradation were examined. As is known, these solar cells can be used in combination with several thin layers of other semiconductors with different bandgaps, such as AlGaAs, InP, GaInP, InGaAs, InGaP, and others. GaAs-based thin-film technology is over 50 years old and constantly evolving. To date, no successful challenger has been found to achieve such a high efficiency, which currently stands at 47.1% with the concentrator. Tripe-junction constructions have become a standard today, but experimentally, there are also constructions with seven or even eight layers. However, the question arises of the technical complexity, price, and meaning of using such a construction. How far can we go?

The solution may be to use different or new, more precise, and less demanding growing manufacturing processes and grown concepts. It has been reported from many publications that the most powerful solar cells use IMM. Another way may be to use concentrators, with which the record, as mentioned above was achieved. For this reason, part of this work was devoted to concentrators, as they are often combined with multilayer GaAs cells. Even here, there is a current development for excellent efficiency, hybridization, or miniaturization.

Miniaturization of concentrators can be used (and already is used) in space technologies, where GaAs cells make the most sense in terms of their good resistance to radiation and their ability to withstand very high-temperature fluctuations. Therefore, it is essential to focus not only on the effectiveness of the PV cell but also on its ability to resist degradation even in inhospitable conditions.

If we summarize the above overview of the past and present state, GaAs solar cells will not have a worthy challenger in many ways for some time to come. However, there are still many reasons to improve and drive their development forward.

## Figures and Tables

**Table 1:** Currently and commonly used materials for III–V compound solar cells, their bandgap $E_{g}$ at 0 K and 300 K, type of the structure (*d* direct or *i* indirect) and the lattice constant at 300 K. Germanium and silicon are also given below for the comparison written in italics [16,17].

Semiconductor	Chemical Formula	Bandgap/eV ( 0 K)	Bandgap/eV ( 300 K)	Gap	Lattice Constant/Å ( 300 K)
Gallium arsenide	GaAs	1.52	1.42	d	5.653
Indium phosphide	InP	1.42	1.35	d	5.869
Gallium antimonide	GaSb	0.81	0.72	d	6.096
*Silicon*	*Si*	*1.17*	*1.12*	*i*	*5.431*
*Germanium*	*Ge*	*0.74*	*0.66*	*i*	*5.658*

**Table 2:** The data below show a significant decrease in power after heating to 350 ${}^{\circ }C$ for 240 min. Selected parameters are open-circuit voltage $V_{\mathrm{oc}}$, short-circuit current $I_{\mathrm{sc}}$, voltage at MPP $V_{\mathrm{mpp}}$, current at MPP $I_{\mathrm{mpp}}$, power at MPP $P_{\mathrm{mpp}}$, and fill factor FF.

	$V_{\mathrm{oc}}$/mV	$I_{\mathrm{sc}}$/mA	$V_{\mathrm{mpp}}$/mV	$I_{\mathrm{mpp}}$/mA	$P_{\mathrm{mpp}}$/mW	*FF*/–
Before processing	783.0	3.190	600.5	2.821	1.694	0.678
After processing	741.8	2.989	480.6	2.300	1.105	0.274

**Table 3:** Electrical parameters from light I–V measurements of the PV cell during the supercontinuum laser processing [73].

Days	$V_{\mathrm{oc}}$/mV	$I_{\mathrm{sc}}$/mA	$V_{\mathrm{mpp}}$/mV	$I_{\mathrm{mpp}}$/mA	$P_{\mathrm{mpp}}$/mW	*FF*/–
0	832.4	8.701	660.6	8.965	5.262	0.727
7	817.5	8.661	650.7	7.803	5.078	0.717
20	810.7	8.851	595.6	7.560	4.503	0.628
32	813.9	8.440	545.5	7.031	3.836	0.558
42	787.7	8.513	580.5	7.432	4.315	0.643
57	750.5	8.687	520.5	7.277	3.787	0.581

## Sample Availability

Samples of single-junction GaAs-based solar cell are available on demand from Nikola Papež. E-mail: Nikola.Papez@vutbr.cz.

## Abbreviations

The following abbreviations are used in this manuscript:

AFM	Atomic Force Microscope
AM0	Air Mass at zero atmosphere
ARC	Anti-Reflection Coating
CPV	Concentrator Photovoltaics
CSOC	Concentrator Standard Operating Conditions
CSTC	Concentrator Standard Test Conditions
EBIC	Electron Beam-Induced Current
HALE	High Altitude Long Endurance
HCPV	High-Concentration Photovoltaics
HEMT	High Electron Mobility Transistor
LCPV	Low Concentration Photovoltaic
MBE	Molecular Beam Epitaxy
MOVPE	Metal Organic Vapor Phase Epitaxy
MPCV	Multipurpose Crew Vehicle
MPP	Maximum Power Point
NASA	National Aeronautics and Space Administration
NREL	National Renewable Energy Laboratory
PV	Photovoltaic
SEM	Scanning Electron Microscope
SOE	Secondary Optical Element
SoG	Silicone-on-Glass
IMM	Inverted Metamorphic
IUPAC	International Union of Pure and Applied Chemistry
IR	Infrared
ISE	Institute for Solar Energy Systems
UAV	Unmanned Aerial Vehicles
UHV	Ultra-High Vacuum
UV	Ultraviolet
